# Stroke as the Sole Manifestation of Takayasu Arteritis in a 15-Year-Old Boy with Latent Tuberculosis

**DOI:** 10.1155/2016/8736248

**Published:** 2016-11-14

**Authors:** Espen Benjaminsen, Anne Reigstad, Vanja Cengija, Vibke Lilleby, Maria Carlsson

**Affiliations:** ^1^Department of Neurology, Nordland Hospital, Bodø, Norway; ^2^Division of Internal Medicine, Nordland Hospital, Bodø, Norway; ^3^Department of Radiology, Oslo University Hospital, Rikshospitalet, Oslo, Norway; ^4^Department of Rheumatology, Oslo University Hospital, Rikshospitalet, Oslo, Norway; ^5^Department of Clinical Medicine, The Arctic University of Tromsø (UiT), Tromsø, Norway

## Abstract

*Introduction*. Takayasu arteritis is a rare disease affecting the aorta and its main branches, causing arterial claudication and end-organ ischemia, including stroke. The etiology is unknown but is believed to be autoimmune. An association between Takayasu arteritis and tuberculosis has been suggested, but the possible relation is unclear.* Case Presentation*. A 15-year-old Somali boy was diagnosed with latent tuberculosis. He had a lesion in the right lung, and both the tuberculin skin test by the Mantoux method and Quantiferon GOLD test turned out positive. After he suffered a cerebral infarct in the right hemisphere, childhood Takayasu arteritis was diagnosed. The diagnosis was based on diagnostic imaging showing a high-grade stenosis of the origin of the right common carotid artery, an occluded common carotid artery on the left side, a circumferential thickening of the vessel walls in the right and left common carotid artery, and laboratory findings with elevated C-reactive protein.* Conclusion*. Takayasu arteritis is an uncommon cause of stroke. It should however be kept in mind as a cause of cerebrovascular disease, especially in the young.

## 1. Introduction

Takayasu arteritis (TA) is a chronic granulomatous inflammation affecting the aorta and its main branches, causing stenosis, dilatation, and aneurisms of the vessels. It is a rare disease with the highest prevalence reported in Asia. A study showed a prevalence of 40 per million in Japan [1]. In the first half of the 1970s, the prevalence in Sweden was 6.4 per million [2]. A study from England reported an annual incidence of 0.8 per million in the period of 2000–2005, with a prevalence of 4.7 per million [3]. The etiology is unknown but is believed to be autoimmune. The clinical symptoms are mainly due to arterial claudication and end-organ ischemia, including stroke, often preceded by night sweat, fatigue, weight loss, myalgia, arthralgia, and malaise [4]. According to the American College of Rheumatology's criteria for the diagnosis of TA, the patient should fulfill at least three of the following six: arteriographic evidence of narrowing or occlusion of the aorta, its main branches, or large arteries in proximal upper or lower extremities, decreased brachial artery pulses, claudication of an extremity, asymmetric (>10 mm Hg) systolic blood pressure in the arms, bruit over the aorta or subclavian arteries, and age at onset < 40 years [5]. However, revised diagnostic criteria for childhood TA were published in 2010 [6]. In children, angiographic abnormality, with aneurism or dilatation, narrowing, occlusion, or thickening of the aorta or its main branches, is a mandatory criterion for the diagnosis. In addition, one of the following should be fulfilled: pulse deficit or claudication, blood pressure discrepancy > 10 mm Hg in any limb, hypertension with systolic or diastolic > 95th centile for height, and erythrocyte sedimentation rate (ESR) > 20 mm/h or elevated C-reactive protein (CRP).

We report a case of a 15-year-old boy with latent tuberculosis (TBC), where ischemic stroke was the sole clinical manifestation of TA.

## 2. Case Presentation

**Figure 1:**
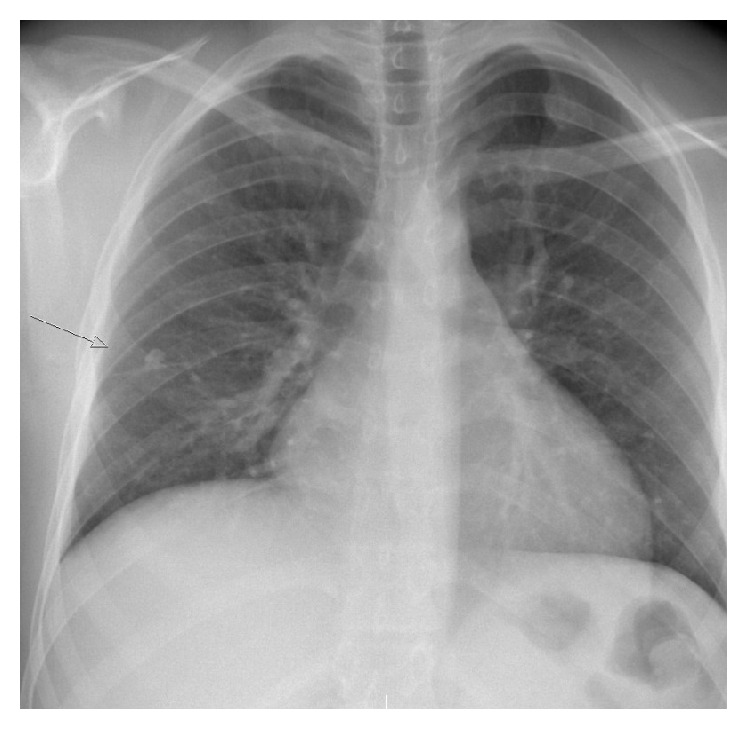
Chest X-ray showing a lesion in the right lung.

A 15-year-old boy originally from Somalia underwent routine TBC screening on arrival in Norway. Half a year earlier, he had a transient episode with malaise and fever, with no identified underlying cause. On the time of the TBC examination, he had no cough, fever, weight loss, or night sweats. Both the tuberculin skin test by the Mantoux method and Quantiferon GOLD test turned out positive. He was not previously vaccinated for TBC. X-ray of the chest revealed a 10 mm lesion in the right lung (Figure 1). There were no acid-fast bacilli detected on microscopic examination of smears from induced sputum, and the culture later proved negative. Pending culture results, he was started on basic TBC treatment with rifampin, isoniazid, pyrazinamide, and ethambutol. After the initiation of treatment, he felt unwell with nausea and anorexia.

**Figure 2:**
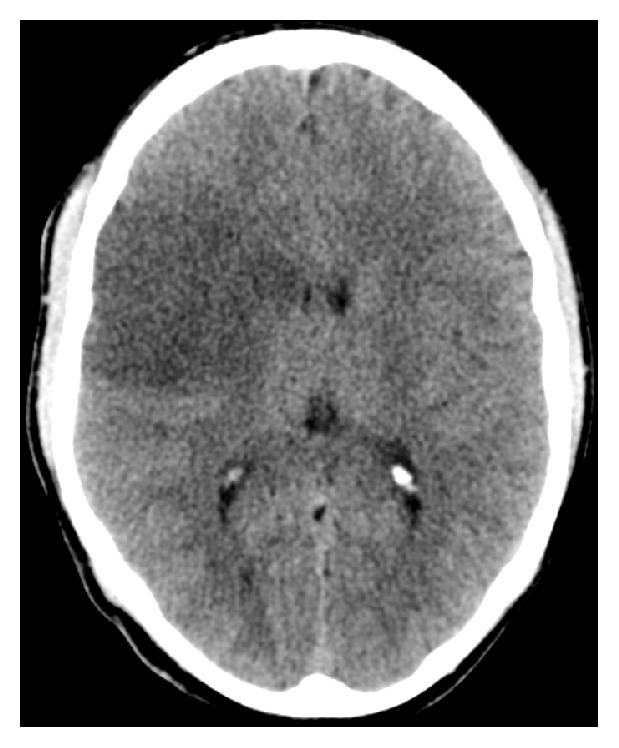
Computed tomography of the brain showing a cerebral infarction in the right hemisphere.

**Figure 3:**
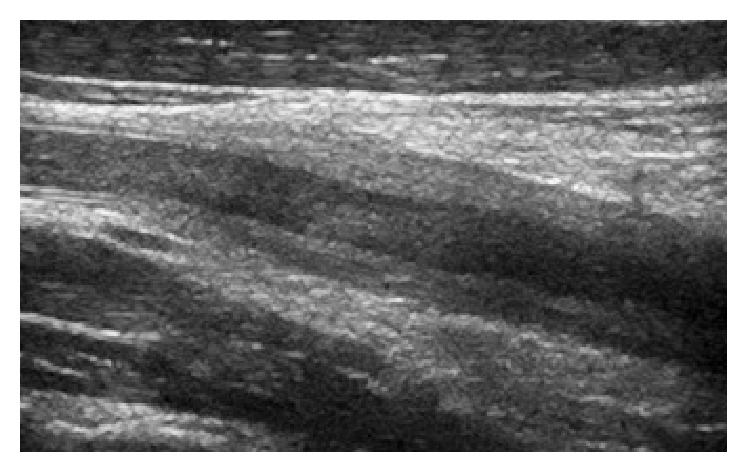
A circumferential thickening of the common carotid artery wall was detected on Doppler ultrasonography, while the vessel wall of the internal carotid artery was normal.

**Figure 4:**
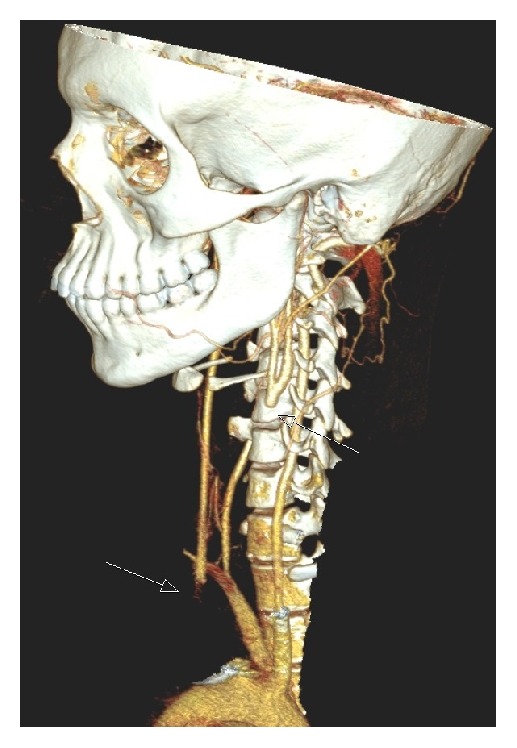
Spiral CT angiography displaying a high-grade stenosis at the origin of the right common carotid artery, whereas the left common carotid artery is occluded.

Fifteen days later, he was admitted to the hospital with acute left hemiparesis. Computed tomography (CT) of the brain showed a cerebral infarction in the right hemisphere (Figure 2). Doppler ultrasonography of the cervical vessels showed a marked, homogenous, circumferential thickening of the vessel walls in the right and left common carotid artery. There was no calcification of the vessel walls. There was a stenosis of the caudal part of the right common carotid artery and a poststenotic flow with low velocities in the medial and cranial part of the vessel, while the left common carotid artery was occluded (Figure 3). The vessel walls were normal in the internal and external carotid arteries on both sides. A retrograde flow was seen in the left external carotid artery, while a slow antegrade flow was detected within the left internal carotid artery. The vertebral arteries were open with high flow velocities, but no signs of stenosis. Spiral CT angiography demonstrated a high-grade stenosis of the origin of the common carotid artery on the right side, whereas the common carotid artery on the left side was occluded (Figure 4). The internal and external carotid arteries appeared normal on both sides. The subclavian and vertebral arteries as well as the arteries of Willis circle had normal contrast enhancement. Echocardiogram and transthoracic and transesophageal echocardiography were normal. There were no bruits over the heart or the neck. The patient had full pulses in the radial arteries, with normal and symmetrical blood pressure in the arms. He had previously suffered a bilateral below-knee traumatic amputation. Due to multiple metal fragments in his stumps, magnetic resonance imaging (MRI) was not performed. During the first week of hospitalization, he complained of pain in his legs. CT of the legs showed no abscesses, and, with the theory of phantom pain, treatment with gabapentin was initiated with good pain relief. Blood tests showed a slightly elevated ESR of 18 mm/h and a CRP of 25 mg/L. The analyses were otherwise normal with respect to hemoglobin, leucocytes and platelets, international normalized ratio (INR), activated partial thromboplastin time (APTT), anti-neutrophil cytoplasmic antibodies (ANCA), rheumatoid factor (RF), antinuclear antibodies (ANA), and anticardiolipin antibodies. Blood culture showed no growth of aerobic or anaerobic bacteria, HIV test was negative, and* Treponema pallidum* was not detected neither in the blood nor in the cerebrospinal fluid. Lumbar puncture showed an opening pressure of 17 cm H_2_O with clear and colorless cerebrospinal fluid, with normal amount of cells counts, total protein, and glucose.

Treatment with aspirin was started at admittance. The raised ESR and CRP in addition to the findings on ultrasonography and CT angiography raised suspicion of vasculitis. Therefore, treatment with prednisolone was also initiated, augmented a few weeks later with methotrexate. One year thereafter, the Doppler ultrasonography findings were unchanged. Two years after the stroke, he still had a hemiparesis but developed no new clinical signs or symptoms.

## 3. Discussion

We present a case of a large ischemic stroke in a 15-year-old boy, with no known risk factors. There were no signs of atherosclerosis and no evidence of developmental abnormalities affecting the aorta such as coarctation or Marfan syndrome. There was affection of the large arteries, thus raising suspicion of large artery vasculitis. The ESR and CRP were elevated, indicating inflammation. The possibility of giant cell arteritis was considered but found unlikely due to the patient's young age with only slight ESR elevation. There were no findings indicating rheumatoid arthritis, systemic lupus erythematosus, or Wegener granulomatosis.

With the characteristic findings on Doppler ultrasonography and CT angiography with stenosis, occlusion, and thickened arterial walls, in addition to blood tests indicating acute phase reaction with elevated CRP, the diagnosis of childhood TA was affirmed [6]. It was classified as type I in accordance with the Hata angiography-based classification [7]. Pathology on angiography is a mandatory diagnostic criterion for childhood-onset TA. Digital suppression angiography is the gold standard, but MR angiography and CT angiography are noninvasive alternatives. Ultrasonography may show a hypoechoic thickening of the vessel walls. Fluorodeoxyglucose positron emission tomography (PDG-PET) may yield diagnostic information about the degree and site of inflammation. Since treatment with steroids was initiated, PDG-PET was not performed in our patient.

Systemic symptoms as fever, weight loss, headache, and raised inflammatory markers are common at TA onset. However, the unspecific nature of the presenting complaints together with the lack of specific disease markers often causes a delay in diagnosis [8]. Therefore, many patients already suffer from symptoms of arterial claudication and end-organ ischemia at the time of diagnosis. These complications, which often are irreversible, include cardiovascular, pulmonary, and neurological complications. The most common cardiovascular complication is hypertension, mainly caused by renal artery stenosis or aortic narrowing and aortic fibrosis. Other cardiovascular complications are myocarditis, coronary artery involvement, aortic regurgitation, aortic aneurysms, and dissection of the aorta. Neurological symptoms include orthostatic hypotension and syncope, headache, and amaurosis fugax [8, 9]. Stroke is a potential serious complication of TA and has been reported as the first manifestation of the disease [10–12]. In a study of 45 patients with TA followed up in a Turkish hospital, cerebrovascular events were the initial complaint in 13% [13], and of 142 patients in a study from South Africa 20% were identified with a primarily cerebrovascular presentation [14].

Our patient had latent TBC, which is a major global health problem, with an estimated 9.27 million new cases in 2007. Stroke in relation to TBC is seen mainly as a consequence of TBC meningitis [15]. Our patient had no visible exudates or meningeal enhancement on CT, and the clinical examination along with the laboratory findings of the blood and CSF gave no hallmarks of TBC meningitis.

Several immune-mediated diseases have been associated with TBC [16], and an association between TA and TBC has been suggested [17, 18]. A possible link could be that a 65 kDa heat shock protein from* M. tuberculosis* (mHSP65) acts as an antigen [19]. It has been suggested that cross reactivity of immune response between mHSP65 and the human heat shock protein-60 could be a possible cause of an autoimmune reaction causing TA [20]. The connection is however uncertain. A study comparing 47 TA patients with TBC and 220 TA patients without TBC did not find any difference in clinical features or angiographic findings between the two groups [21]. One study did not show any difference on Quantiferon test positivity comparing 94 TA patients with 107 controls [22]. Another study did not find any trace of* M. tuberculosis* in the artery walls of 10 patients with TA [23]. However, one study has identified the presence of gene sequences associated with* M. tuberculosis* and* M. bovis* in aorta tissue in patients with TA, and the authors suggested that TA may result from a latent infection with* M. tuberculosis* [24].

## 4. Conclusion

TA, especially with stroke as the first presentation of the disease, is uncommon. It should however be kept in mind as a cause of cerebrovascular disease, especially in the young. In the age of globalization and increased migration, the condition could become more common even in the northwestern corner of Europe.
